# Combination immunotherapy with Interleukin-2 and CTLA-4 blockade decreases tumor growth and improves overall survival

**DOI:** 10.1186/2051-1426-1-S1-P70

**Published:** 2013-11-07

**Authors:** Joseph R Broucek, Tasha Hughes, Erica J Huelsmann, Joseph L Poshepny, Carl E Ruby, Andrew Zloza, Howard L Kaufman

**Affiliations:** 1 Department of Surgery, Rush University Medical Center, Chicago, IL, USA; 2 Department of Immunology/Microbiology, Rush University Medical Center, Chicago, IL, USA; 3 Department of Internal Medicine, Rush University Medical Center, Chicago, IL, USA

## Background

Combination immunotherapy is quickly gaining attention in the field of cancer treatment. IL-2, a cytokine which activates T cells, and ipilimumab, a monoclonal antibody that blocks CTLA-4, are both approved as monotherapy for metastatic melanoma. To date, combination immunotherapy with IL-2 and anti-CTLA-4 has not been adequately investigated. We hypothesized that that this combination may work synergistically owing to its distinct but complementary mechanisms of T cell activation.

## Methods

C57BL/6 mice (10 per group) were challenged with B16-F10 melanoma (via intradermal injection with 120,000 cells) on day 0. Four groups were treated via intraperitoneal injection: -Group 1 (combination): IL-2 (100,000 units in 100 µl) every 12 h on days 4-8 and anti-CTLA-4 (100 µg in 100 µl) on days 3, 6, and 9; -Group 2 (IL-2 only): IL-2 (100,000 units in 100 µl) every 12 h on days 4-8 and IgG (100 µg in 100 µl) on days 3, 6, and 9; -Group 3 (anti-CTLA-4 only): PBS (100 µl) every 12 h on days 4-8 and anti-CTLA-4 (100 µg in 100µl) on days 3, 6, and 9; -Group 4 (placebo): PBS (100 µl) every 12 h on days 4-8 and IgG (100 µg in 100 µl) on days 3, 6, and 9. Tumor area (length*width) was measured every other day until death of the animal or until tumors reached 100mm2, when animals were sacrificed as per institutional protocols. Primary outcomes included tumor size and overall survival.

## Results

Tumor growth was significantly reduced with combination IL-2 and anti-CTLA-4. Specifically, on day 14, the mean tumor area with combination IL-2 and anti-CTLA-4 was 2mm2, while in the anti-CTLA-4 only, IL-2 only, and placebo groups it was 14, 29, and 68 mm2, respectively (P<0.01 for all comparisons, except IL-2 only versus anti-CTLA-4 only [not significant]). At day 30, the overall survival with combination IL-2 and anti-CTLA-4 was 50%, while with IL-2 only and anti-CTLA-4 only it was 10% and 20%, respectively (P<0.01 for all comparisons). All animals treated in the placebo group succumbed to their tumors by day 19. These findings were confirmed in a second experiment with similar results.

## Conclusions

Combination immunotherapy with IL-2 activation and CTLA-4 blockade significantly decreases tumor growth and increases overall survival when compared to IL-2 or anti-CTLA-4 monotherapy or placebo. The role of CD8+ effector versus CD4+ regulatory T cells in the success of this immunotherapy is ongoing and will be included in our formal presentation. Ultimately, we aim to translate this work into a combination immunotherapy clinical trial.

